# Hybrid Silica-Phytic Acid Coatings: Effect on the Thermal Stability and Flame Retardancy of Cotton

**DOI:** 10.3390/polym11101664

**Published:** 2019-10-12

**Authors:** Marco Barbalini, Luca Bertolla, Jaromír Toušek, Giulio Malucelli

**Affiliations:** 1 Department of Applied Science and Technology, Local INSTM Unit, Viale Teresa Michel 5, 15121 Alessandria, Italy; marco.barbalini@polito.it; 2 Institute of Physics of Materials of the Czech Republic, Brittle Fracture Group, Žižkova 22, 61662 Brno, Czechia; bertolla@drs.ipm.cz; 3 CEITEC—Central European Institute of Technology, Masaryk University, Kamenice 5/4A, 62500 Brno, Czechia; jaromir.tousek@ceitec.muni.cz

**Keywords:** sol–gel processes, flame retardancy, cotton, phytic acid, solid state NMR spectroscopy, thermal stability, durability

## Abstract

New hybrid sol–gel coatings based on tetraethoxysilane (TEOS) and phytic acid (PA) were designed and applied to cotton; the flame-retardant properties of the treated fabrics were thoroughly investigated by means of flame-spread and forced-combustion tests. The first goal was to identify the TEOS:PA weight ratio that allowed the achievement of the best flame-retardant properties, with the lowest final dry add-on on the fabrics. Therefore, different TEOS:PA sols were prepared and applied to cotton, and the resulting coated fabrics were thoroughly investigated. In particular, solid-state NMR spectroscopy was exploited for assessing the condensation degree during the sol–gel process, even for evaluating the occurrence of possible reactions between phytic acid and the cellulosic substrate or the alkoxy precursor. It was found that a total dry add-on of 16 wt % together with 70:30 TEOS:PA weight ratio provided cotton with self-extinction, as clearly indicated by flame-spread tests. This formulation was further investigated in forced-combustion tests: a significant reduction of heat release rate (HRR), of the peak of HRR, and of total heat release (THR) was found, together with a remarkable increase of the residues after the test. Unfortunately, the treated fabrics were not resistant to washing cycles, as they significantly lost their flame-retardant properties, consequently to the partial removal of the deposited hybrid coatings.

## 1. Introduction

In the last century, polymers and cellulosic textiles became two of the most universally used but also the most combustible materials [1,2]. In fact, cellulosic materials, like cotton, flax, and hemp, show low fire resistance and burn easily when put in contact with a flame or exposed to a heat source. At high temperatures, these materials produce a wide range of volatile organic compounds that are highly flammable; when exothermic oxidation of the volatiles occurs, these materials definitely burn.

A possible solution to enhance the level of fire safety of combustible fibers and fabrics refers to the use of flame-retardant (FR) chemicals, designed to minimize the rate of flame spread and to prevent sustained combustion [3,4]. In this context, in the last decades, different FRs were developed; these compounds may contain halogens (chlorine and bromine), phosphorus, magnesium, nitrogen, aluminum, antimony, molybdenum, or quite recently developed nanofiller-based systems [5,6,7]. In particular, though halogen-based compounds are the most widely used FRs, and they were proved to be persistent, bioaccumulative, and/or environmentally toxic for both animals and humans [8,9]. Recently, some studies were carried out to develop possible alternatives with a low-environmental impact: in this context, biomacromolecules, including nucleic acids, proteins, pomegranate-rind extracts, and banana pseudostem sap, were thoroughly investigated [10,11,12,13,14,15,16,17,18]. Thanks to their chemical structures and composition, these biomacromolecules show interesting flame-retardant features when applied to cotton fabrics. In particular, it was proved that the phosphorus-based biomacromolecules can act in condensed phase by favoring the formation of stable aromatic char; furthermore, some of them can be considered to be intumescent-like compounds [14,19,20,21].

**Figure 1:**
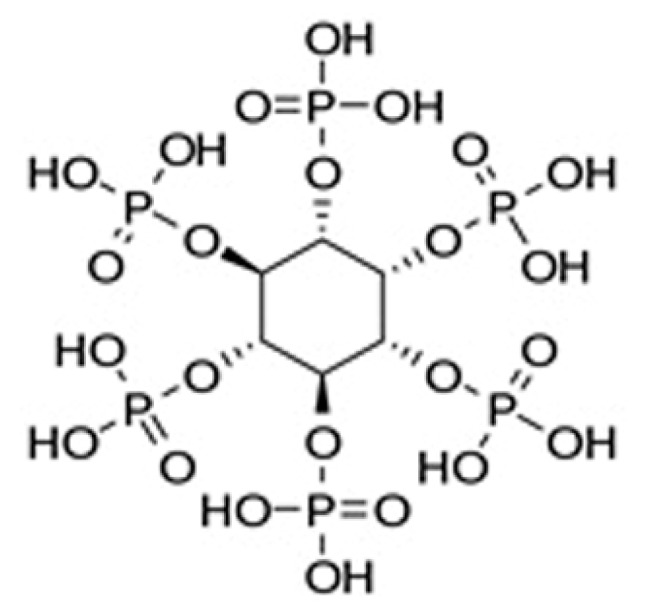
Chemical structure of phytic acid.

In this context, phytic acid, a naturally occurring molecule, is one of the major storage forms of phosphorus-containing compounds; its green character is related to the possibility of being extracted from different plant tissues, such as soy beans, cereal grains, and oil seeds [22]. This eco-friendly, biocompatible and nontoxic organic polyphosphoric acid was employed for specific applications; in particular, it was exploited as a flame-retardant for different textiles, like poly(lactic acid) nonwoven fabrics [23], silk [24], wool [25], and cotton fabrics [26,27], showing good fire performances. In addition to its high phosphorous content (28 wt % of phosphorus based on molecular weight), its special chemical structure (Figure 1) can be potentially used for designing an effective flame-retardant finishing for cellulosic materials. In fact, its structure consists of 6 phosphate groups that, upon exposure to a flame or a heat flux, may give rise to the formation of phosphoric acid; this latter can act in condensed phase, favoring the dehydration of the cellulosic substrate and subsequently forming a stable protective char, hence effectively affecting combustion processes [28].

Sol–gel processes starting from different precursors are widely employed for the design of flame-retardant coatings in situ created on cotton, polyester, and their blends. The resulting ceramic phases are able to effectively protect the underlying substrates from the exposure to a flame or a heat flux [29].

To the best of our knowledge, the effect of the presence of phytic acid in a silica coating deposited on cotton fabrics through a sol–gel process was not investigated so far. According to the chemical structure of PA, it seemed reasonable to assess whether the phosphate groups of phytic acid may take part in the sol–gel process, either reacting with the partially hydrolyzed alkoxy groups of the sol–gel precursor or with the hydroxyls of the cellulosic substrate. This way, the combination of silica and PA in a hybrid coating may result in a durable treatment for cotton, providing, at the same time, an acceptable flame-retardant behavior.

Therefore, in this work, we first identified the best TEOS:PA weight ratio that allowed achieving self-extinction in both horizontal and vertical flame-spread tests, keeping, at the same time, the lowest final FR dry add-on on the treated cotton fabrics. The morphology of the latter was characterized by means of FESEM and FTIR–ATR spectroscopy measurements; furthermore, the condensation degree of the sol–gel precursor and the possible involvement of phytic acid in the sol–gel reactions were investigated by solid-state ^29^Si and ^31^P NMR analyses. Thermogravimetry and flame-spread tests (carried out in horizontal and vertical configuration) were exploited for assessing the thermal and thermo-oxidative behavior and the flame-retardant properties provided by the deposited coatings. Then, the most FR performing system in flame-spread tests was further investigated as far as its behavior to forced combustion is concerned. Finally, the durability (i.e., washing fastness) of the proposed flame-retardant treatment was evaluated, comparing the flame retardancy of the washed treated fabrics with the pristine counterparts.

## 2. Materials and Methods

### 2.1. Materials

Knitted pure cotton fabrics (220 g/m^2^ and 0.2 mm thick) were purchased from Fratelli Ballesio S.r.l. (Torino, Italy).

Phytic acid (PA, as 50 wt % aqueous solution) was purchased from Tokyo Chemical Industry Co., Ltd. (Tokyo, Japan) and used as received.

Tetraethoxysilane (Si(OCH_2_CH_3_)_4_; purity >99.0 wt %), dibutyltin diacetate (C_12_H_24_O_4_Sn, used as condensation catalyst) and ethanol (employed as co-solvent in the preparation of the sols) were purchased by Aldrich Chemical Co. (Milano, Italy) and used as received.

### 2.2. Preparation of the Sols

The sols were prepared by mixing phytic acid and TEOS in the desired weight ratios and adding one drop of dibutyltin diacetate as condensation catalyst. The mixtures were kept under mechanical stirring, while ethanol was added drop-wise, at room temperature, in order to favor the dissolution of TEOS in water. Thanks to the water content of the phytic acid solution, the hydrolysis reaction involved in sol–gel process did not require a further addition of water; the intrinsic acidic character of phytic acid was exploited, avoiding the use of acidic catalysts (like HCl) for performing the sol–gel process. The recipes of the designed and prepared sols are collected in Table 1.

### 2.3. Sol–Gel Treatments Performed on Cotton

Cotton fabrics were cut into square pieces (10 × 10 cm), weighted, and then impregnated with the sols. Furthermore, two cotton fabrics were impregnated with phytic acid (sample name: COT + PA) or TEOS only (sample name: COT + TEOS, Table 1).

The impregnated fabrics were put on a glass substrate and thermally treated in an oven at 80 °C for 1 h and 30 min, in order to perform the sol–gel reactions. Then, the final dry add-on on the cotton samples (i.e., the dry weight gain (A), wt %) was determined by weighing each sample before (Wi) and after the impregnation with the sol and the subsequent thermal treatment (Wf). The weight gain of the treated fabric was calculated using the following formula:

$$A=\frac{\mathrm{Wf}-\mathrm{Wi}}{\mathrm{Wi}}\times 100$$

It is worthy to note that, as far as COT + TEOS and COT + PA samples are considered, it was not possible, even exploiting multiple impregnation steps, to overcome 10 and 15 wt % of dry add-on for the two systems, respectively. Therefore, they were utilized as reference systems and compared with the fabrics treated with the hybrid coatings.

### 2.4. Characterization Techniques

The morphology of treated and untreated cotton fabrics was investigated using a FESEM (ZEISS, MERLIN) apparatus. Cotton fabrics were cut and metallized with platinum (thickness of the layer: 5 nm).

A Perkin Elmer Spectrum 100 spectrometer (Shelton, Connecticut, USA) equipped with an attenuated total reflection (ATR) diamond accessory was employed for collecting the FTIR spectra of untreated and treated cotton. FTIR spectra were recorded at wavelengths from 700 to 4000 cm^−1^ with 4 cm^−1^ resolution; 16 scans were collected.

Then, ^31^P and ^29^Si NMR spectra were obtained using a Bruker Avance-500 spectrometer (Ettlingen, Germany), operating at frequencies of 202.49 and 99.38 MHz, respectively. A Bruker 4 mm CP/MAS probe (Ettlingen, Germany) was used for all measurements. Samples were loaded in 4 mm zirconia rotors. The chemical shifts were referenced to NH_4_H_2_PO_4_ (0.8 ppm) for ^31^P and 4,4-dimethyl-4silapentane-1-sulfonic acid (DSS) (0.0 ppm) for ^29^Si. A rotation frequency of 10 kHz was used for all samples. Recycle delay of 2 s (256 accumulations) was used for ^31^P and 20 s (8000 accumulations) for ^29^Si.

The thermal and thermo-oxidative stability of the fabrics was evaluated by thermogravimetric (TG) analyses in nitrogen and air, respectively, from 50 to 700 °C, with a heating rate of 20 °C/min. A TAQ500 analyzer (TA Instrument Inc., Waters LLC, New Castle, Delaware USA) was used, placing the samples (approximately 8 mg) in open alumina pans, in an inert or oxidative atmosphere (gas flow: 35 mL/min).

Horizontal and vertical flame-spread tests were carried out on untreated cotton and on the sol–gel treated fabrics according to UL94 standard. Cone calorimetry tests were performed according to the ISO 5660 standard. In particular, square specimens (10 × 10 cm) were irradiated with a heat flux of 35 kW/m^2^ in horizontal configuration; the fabrics were placed on a sample holder and maintained in the correct position using a metallic grid. For each formulation, the test was repeated three times and the results averaged. A standard deviation of 2% was calculated for the following parameters: time to ignition (TTI, s), total heat release (THR, MJ/m^2^), peak of heat release rate (pkHRR, kW/m^2^), total smoke release (m^2^/m^2^), total smoke production (m^2^), and specific extinction area (m^2^/kg). The residues at the end of the tests were also evaluated.

The washing fastness of the treated fabrics was determined following the AATCC test method 61 (2A)–1996 in the presence of non-ionic detergent at 38 ± 3 °C.

## 3. Results and Discussion

### 3.1. FTIR–ATR Spectroscopy

**Figure 2:**
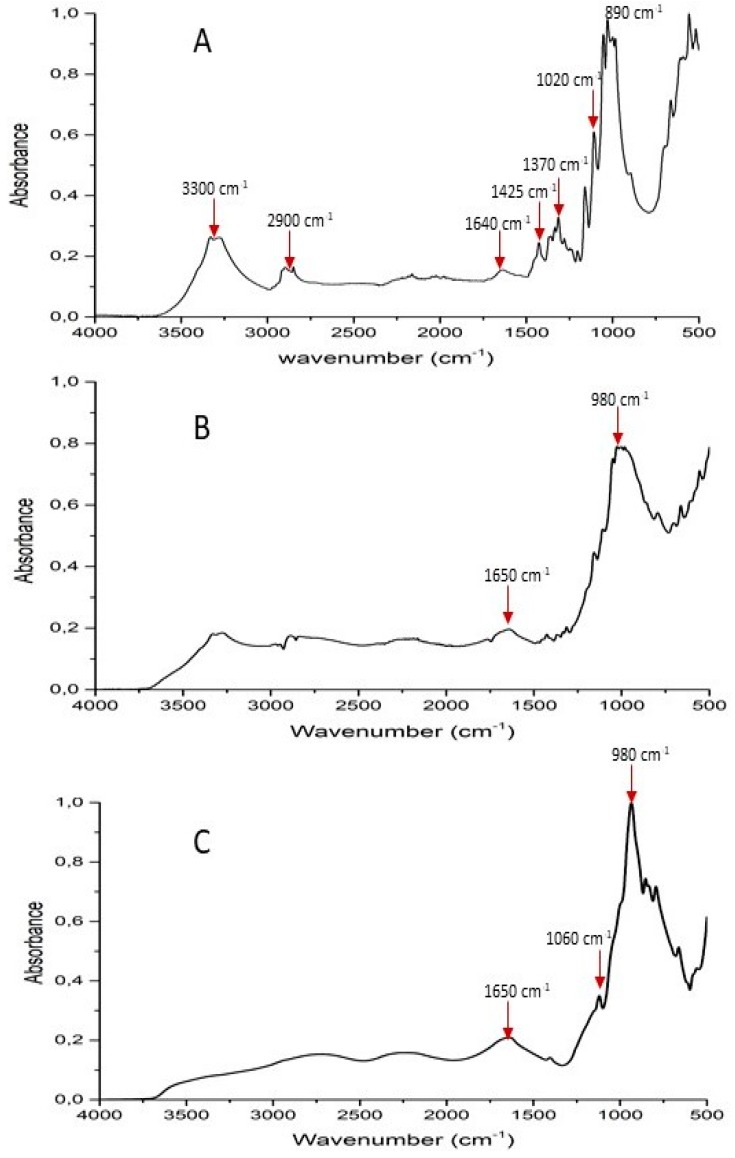
FTIR–ATR spectra of COT (**A**), COT + PA (**B**), and PA (**C**).

**Figure 3:**
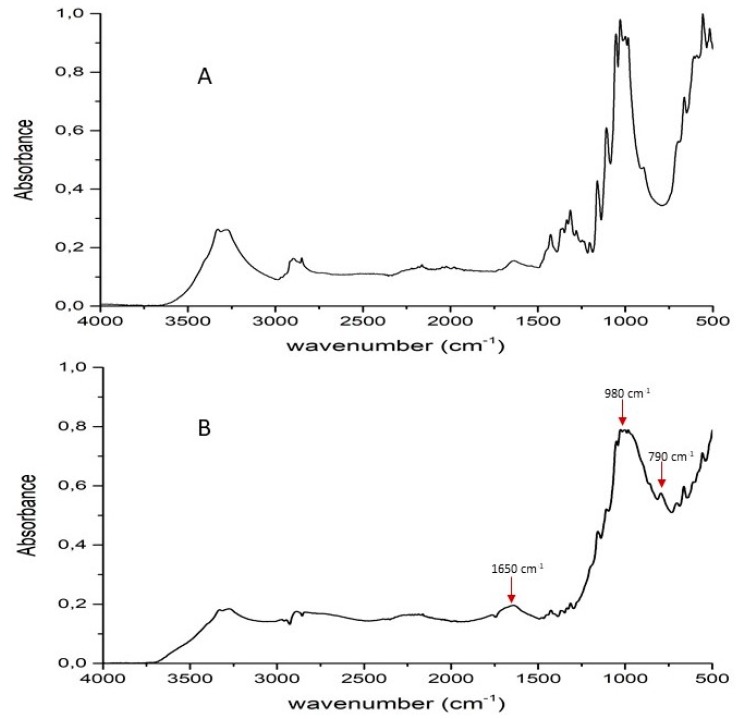
FTIR–ATR spectra of COT (**A**) and COT + SOL (70/30) (**B**).

The effectiveness of the deposition of the sol–gel coatings on the cotton fabrics was assessed through FTIR–ATR spectroscopy. Figure 2 shows the FTIR–ATR spectrum of phytic acid: three characteristic peaks, located at 1650, 1060, and 980 cm^−1^, and corresponding to stretching vibration of P = O, asymmetric and symmetric stretching of P–O–C, are present [30]. Figure 3 compares the typical FTIR–ATR spectra of cotton and of COT + SOL (70/30). The characteristic peaks of cellulose are easily detectable in untreated cotton (namely: *v*(OH) at about 3300, *v*(CH_2_) at 2900, δ (OH) at 1640, δ (CH_2_) at 1425, δ (CH) at 1370, δ (OH) at 1310, *v* (C–C) at 1020, and δ (OH) at 894 cm^−1^). The FTIR–ATR spectrum of cotton treated with the hybrid coating still shows the presence of some typical vibrational modes of cellulose, though these signals are less intense and defined because of the presence the sol–gel coating. Besides, some weak signals referable to the presence of the phytic acid are evident at 1650 and 980 cm^−1^. Finally, the treated cotton shows a peak at 790 cm^−1^, attributable to symmetrical stretching of Si–O–Si; this evidence qualitatively confirms the occurrence of the sol–gel process, with the formation of a ceramic silica phase within the fabric.

### 3.2. Solid State NMR Analysis

**Figure 4:**
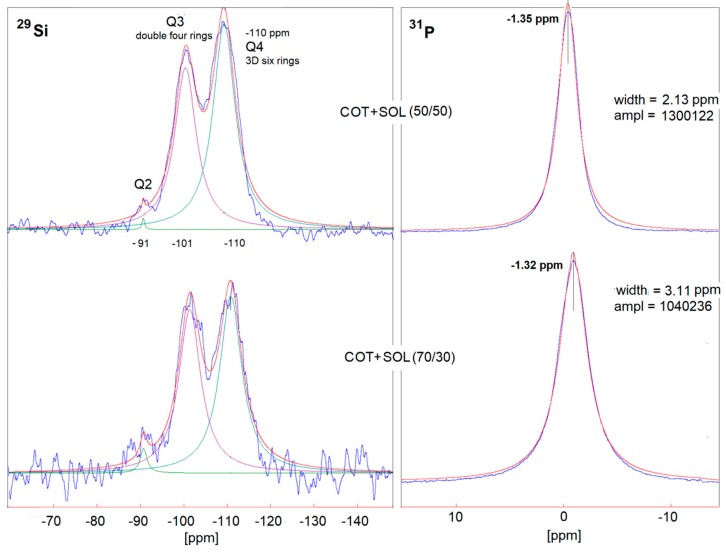
Solid-state ^29^Si (left) and ^31^P (right) NMR spectra of COT + SOL (50/50) and COT + SOL (70/30). (Q2 silica species refers to a central silicon atom connected to 2 silicon species, Q3 refers to a central silicon atom connected to 3 silicon species and Q4 is a central silicon atom connected to four silicon species).

In Figure 4, the typical solid-state ^29^Si (left) and ^31^P (right) NMR spectra obtained for some treated fabrics are shown. The ^29^Si spectra were deconvoluted in three main peaks, centered at approximately −91, −101, and −110 ppm and associated with Q^2^ species (or double three-rings Q^3^), Q^3^ double four-rings, and Q^4^ (3D six-rings), respectively [31]. Nevertheless, in any of the investigated samples, the peak, at approximately −215 ppm (previously assigned to [SiO_6_] units [32] and identifying the transition from the archetypal silicate structure, where all the silicon is tetrahedrally coordinated, to one with octahedrally coordinated silicon), is not detected, thus excluding the formation of Si–O–P bonds, which are generally formed at nearly 400 °C [33]. Conversely, the ^31^P NMR spectra are characterized by a sharp peak centered at approximately a –1.3 ppm, which is typical for mono-phosphate esters. No other peaks are observed, corroborating the hypothesis that the phosphate units are not chemically bonded to each other or to the silicate units [33].

### 3.3. Morphological Analysis

**Figure 5:**
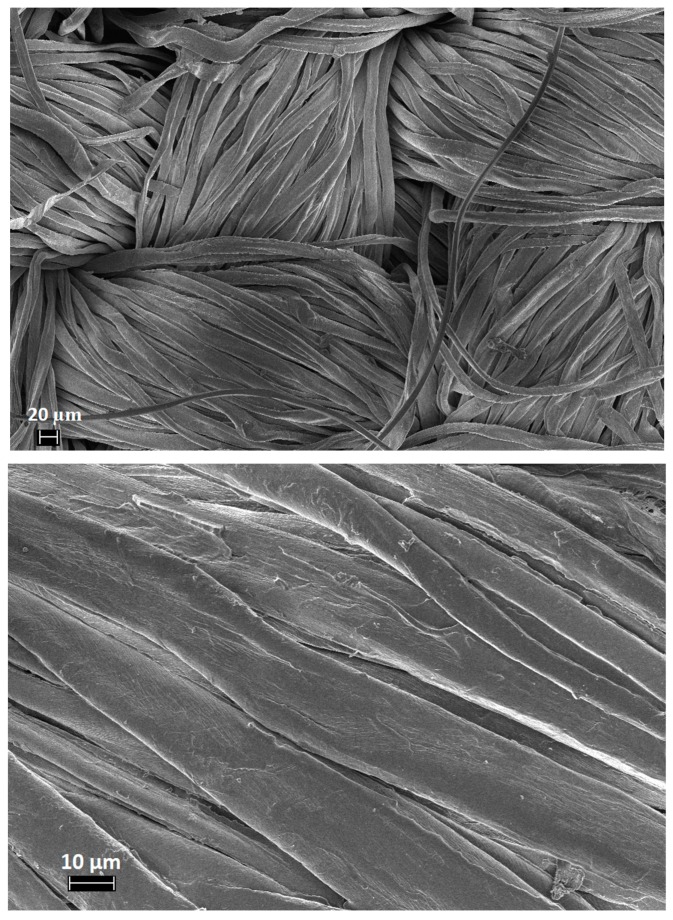
FESEM images of untreated cotton.

**Figure 6:**
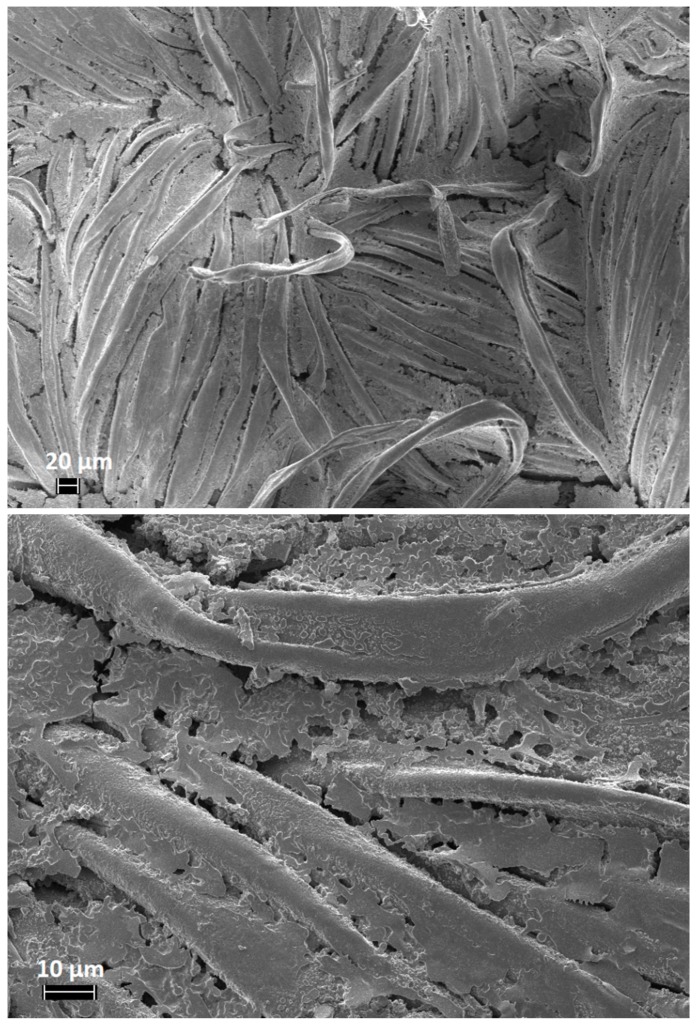
FESEM images of COT + SOL (70/30).

FESEM observations were performed in order to assess the morphology of the cotton fabrics before and after the sol–gel treatments. Untreated cotton shows the characteristic smooth texture (Figure 5); conversely, the treated cotton fabrics (Figure 6 shows the typical images of COT + SOL (70/30)) increase their roughness, due to the presence of the sol–gel coating. Furthermore, it is noteworthy that the sol–gel coating is also present within the fiber interstices.

### 3.4. Thermogravimetric Analysis

**Figure 7:**
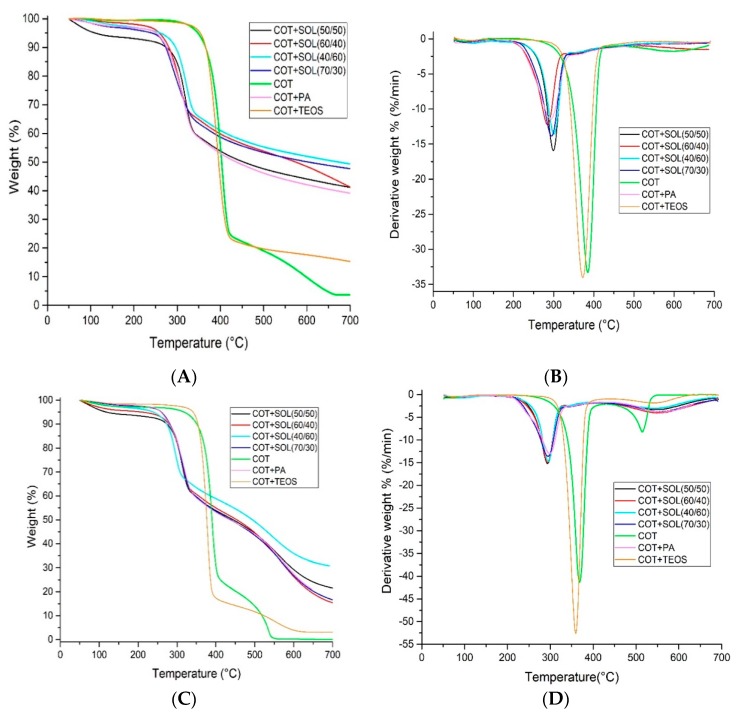
TG curves in N_2_ (**A**) and air (**C**), and dTG curves in nitrogen (**B**) and air (**D**) of cotton and treated cotton fabrics.

TGA was employed for evaluating the thermal and thermo-oxidative stability of untreated and treated cotton fabrics. *T*_ONSET_, the maximum weight loss temperature (*T*_max1_ and *T*_max2_), the residue at the maximum weight loss temperature, and the final residue at 700 °C are collected in Table 2 and Table 3. Figure 7(A–D) shows the corresponding TG and dTG curves. In inert atmosphere (nitrogen), the decomposition of the untreated cotton takes place according to a single main degradation step; the degradation onset occurs at about 358 °C, and the maximum degradation rate is observed at 386 °C (Table 2). The sol–gel coating is responsible for an anticipation of the cellulose decomposition temperature, as revealed by *T*_max_ and *T*_ONSET_, which are shifted toward lower values. These shifts are more pronounced when phytic acid is present in the deposited coating, as it starts decomposing prior to the decomposition of the cellulosic substrate. This finding was already found for cotton fabrics treated with biomacromolecules [14,21]: In doing so, the flame-retardant coating activates, hence favoring the formation of a stable residue (char), able to act as a thermal barrier, instead of the formation of volatile combustible species that could further promote the degradation of the cellulosic substrate [28]. This effect is also very noticeable when cotton is treated with phytic acid only (COT + PA sample, for which the residue at 700 °C approaches 37%). From an overall point of view, the final residue for all the treated fabrics is remarkably higher with respect to the untreated counterpart, again confirming the protection exerted by the deposited hybrid coatings.

In air, cotton decomposition usually occurs by three steps [28]. The first (within 300 and 400 °C) involves two competitive pathways, which produce aliphatic char and volatile products. During the second step (within 400 and 800 °C), some aliphatic char converts into an aromatic form, producing CO and CO_2_ as a consequence of simultaneous carbonization and char oxidation. In the third step (between 700 and 800 °C), the char and hydrocarbon species are further oxidized mainly to CO_2_ and CO.

Table 3 collects the thermogravimetric data in air for cotton and the treated fabrics. Figure 7C,D shows the corresponding TG and dTG curves. In particular, two decomposition peaks (at 368 and 514 °C) are observable for untreated cotton. As already shown in inert atmosphere, irrespective of its composition, the presence of the coating anticipates the decomposition of the cellulosic substrate (*T*_ONSET_ values shift toward lower temperatures, as compared to untreated cotton) and the maximum weight loss (*T*_max1_), but favors the formation of a stable char, as revealed by *T*_max2_ and the final residues, which significantly increase in the sol–gel treated fabrics. In particular, in the hybrid coatings, this finding can be attributed to the decomposition of phytic acid, which favors the dehydration of the underlying cotton substrate, hence the formation of a protective char layer, rather that the development of combustible volatiles.

### 3.5. Fire Behavior

#### 3.5.1. Flame-Spread Tests

**Figure 8:**
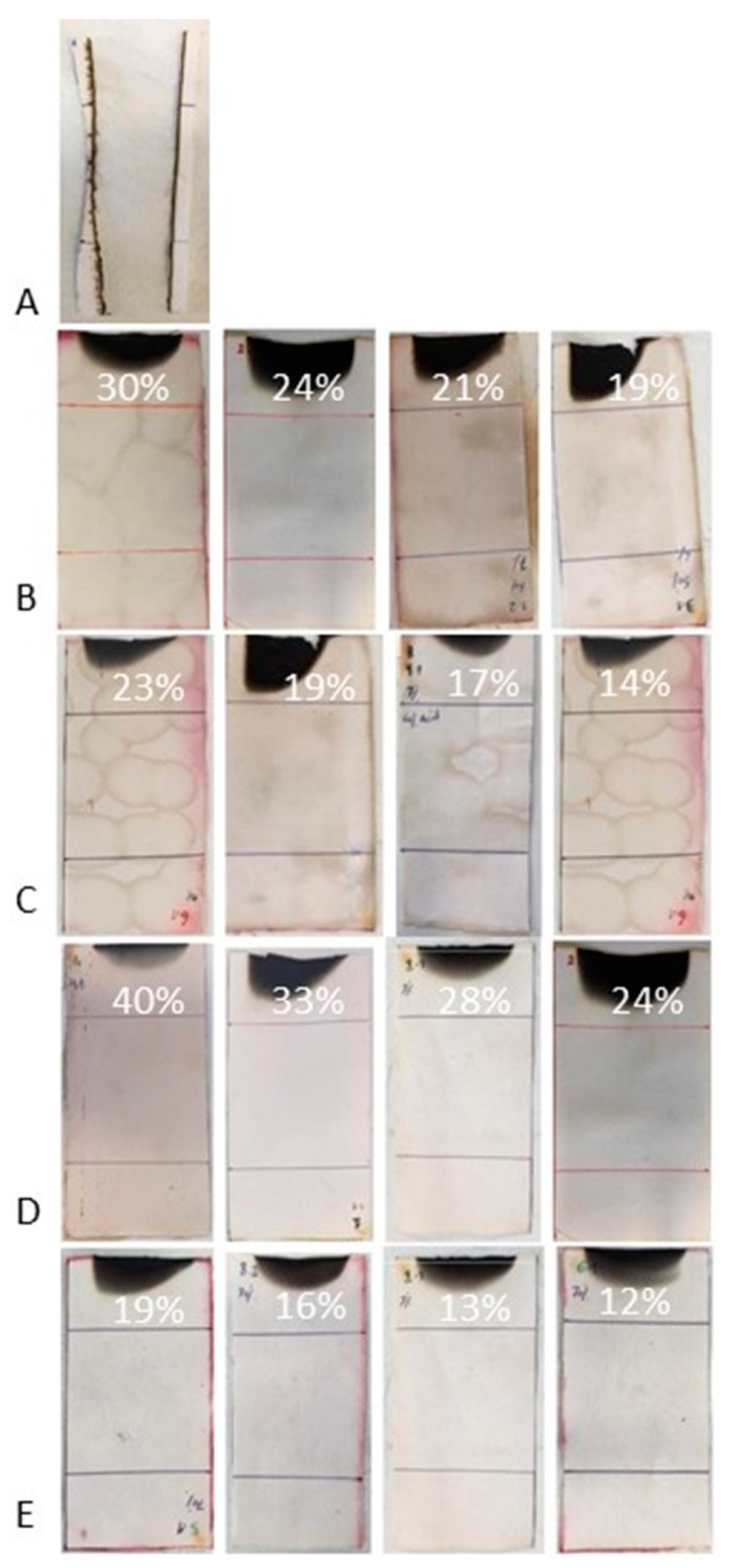
Residues after horizontal flame-spread tests. (**A**) COT, (**B**)COT + SOL (50/50), (**C**) COT + SOL (60/40), (**D**) COT + SOL (40/60), and (**E**) COT + SOL (70/30). The dry add-ons are reported on each image.

**Figure 9:**
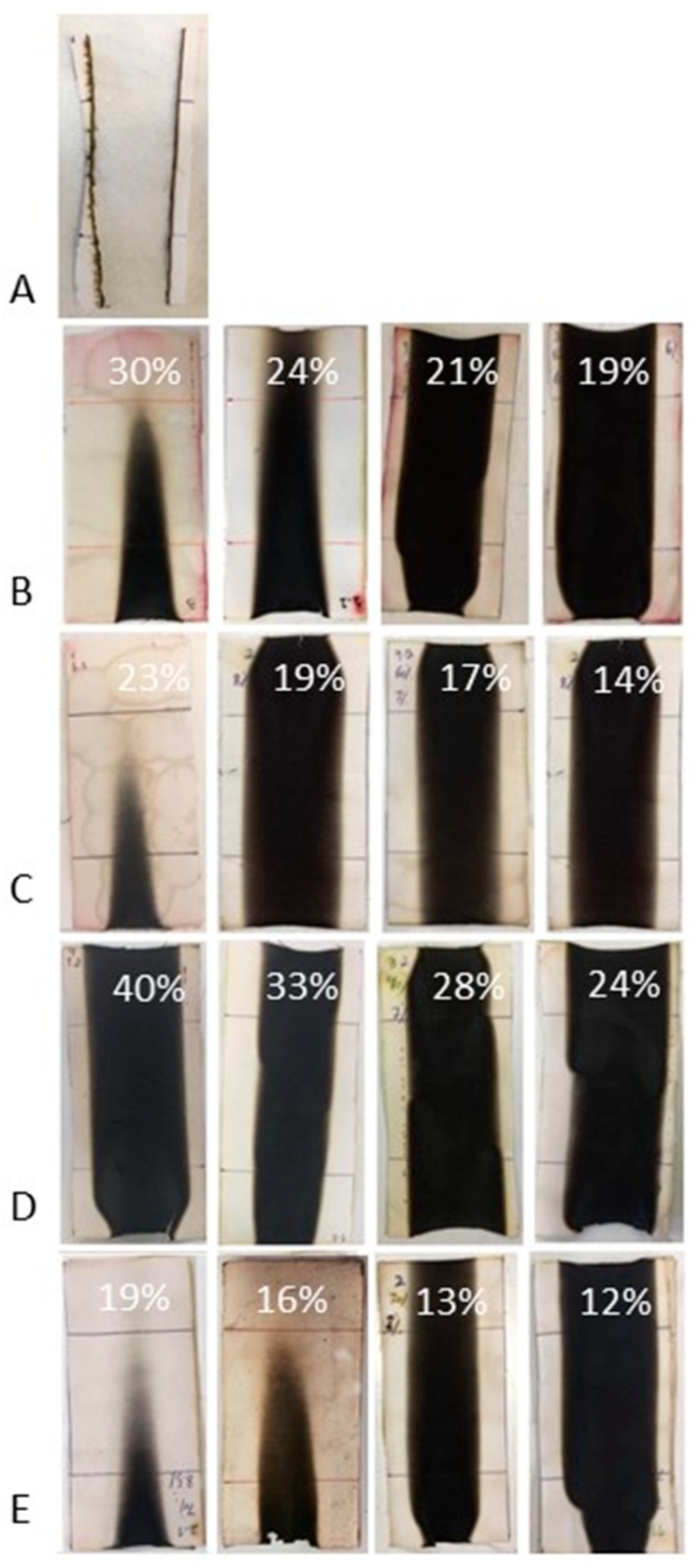
Residues after vertical flame-spread tests. (**A**) COT, (**B**) COT + SOL (50/50), (**C**) COT + SOL (60/40), (**D**) COT + SOL (40/60), and (**E**) COT + SOL (70/30). The dry add-ons are reported on each image.

The flammability of treated and untreated cotton was evaluated by horizontal and vertical flame-spread tests. The results are collected in Table 4 and Table 5; the residues of the fabrics after flammability tests are shown in Figure 8 and Figure 9.

When flammability tests are performed in horizontal configuration, the fabrics treated either with phytic acid only or with the hybrid coatings are all self-extinguishing; conversely, cotton burns very quickly, leaving a negligible residue. The treatment with TEOS only (COT + TEOS sample) does not provide self-extinction, though the burning rate is lower and the final residue is significantly higher with respect to the untreated cellulosic textile. In vertical flame-spread tests (which are more severe than the horizontal ones), the treatment with phytic acid or TEOS only does not allow the achievement of self-extinction; instead, this is fulfilled only with some of the tested sol–gel derived coatings and, in particular, when the highest dry add-ons are applied to cotton. However, for all the hybrid systems, the residues after the vertical flame-spread tests are remarkably higher as compared to cotton, COT + PA, and COT + TEOS systems.

On the basis of these results, we identified COT + SOL (70/30)—with a dry add-on of 16%—as the best-performing system in vertical flame-spread tests (with the minimum FR loading) and, therefore, we decided to further investigate it by means of forced-combustion tests, as it will be detailed in the next paragraph.

#### 3.5.2. Forced-Combustion Tests

**Figure 10:**
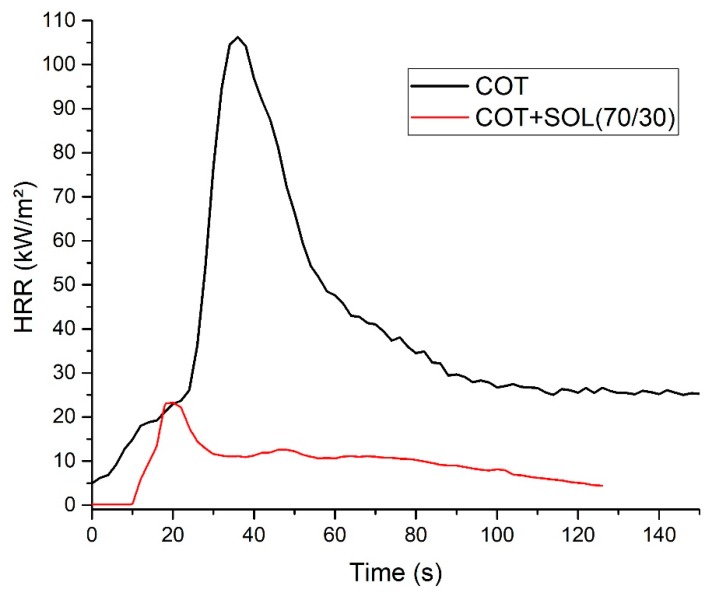
HRR vs. time curves for COT and COT + SOL (70/30).

**Figure 11:**
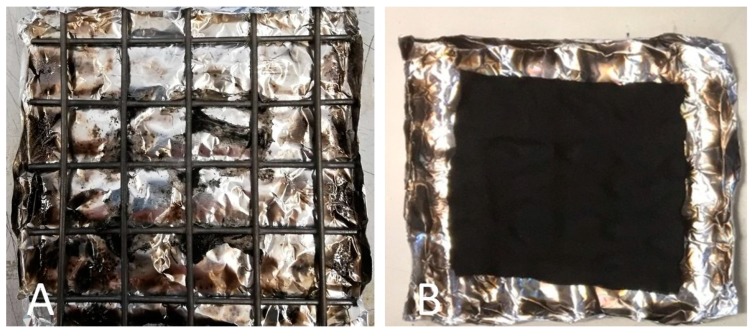
Residues of COT (**A**) and COT + SOL (70/30) (**B**) after cone calorimetry tests.

**Figure 12:**
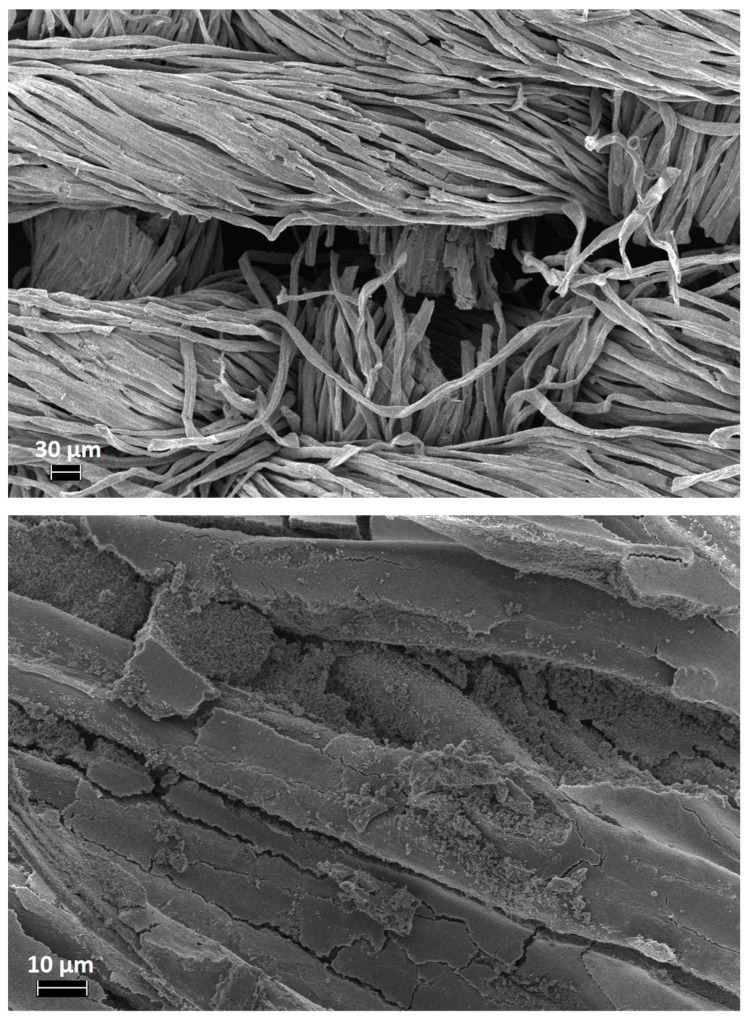
FESEM images of COT + SOL (70/30) after cone calorimetry tests.

To better simulate a realistic fire scenario, cotton and COT + SOL (70/30) with an add-on of 16% were subjected to cone calorimetry tests performed at 35 kW/m^2^. The results are collected in Table 6 (thermal parameters) and Table 7 (smoke parameters); Figure 10 shows the HRR vs. time curves. It is noteworthy that the presence of the hybrid coating significantly affects the forced-combustion behavior of cotton. In particular, the coating turns out to anticipate the ignition of the treated fabric. As already commented in thermogravimetric analyses, this finding can be ascribed to the activation of the hybrid coating (and, in particular, to the phytic acid), which has to decompose prior to the decomposition of the cellulosic substrate. Nonetheless, HRR (mean), pkHRR, and THR values are outstandingly decreased with respect to untreated cotton, by about 36%, 74%, and 39%, respectively. At the same time, the protection exerted by the deposited hybrid coating is witnessed by the important increase of the residue after the test (+25.8%), which is coherent and consistent. The images of the residues after cone calorimetry tests of cotton and the treated counterpart are shown in Figure 11. Figure 12 presents the typical FESEM images of the fabric treated with the hybrid coating at two different magnifications. It is worth noting that the fabric still maintains its texture and the fibers are surrounded by a silica/carbonaceous structure derived from the decomposition of the treated textile during the forced-combustion test.

Another important ability that the designed sol–gel coating should possess refers to the reduction of the smoke parameters. As shown in Table 7, TSR is greatly lowered (−29%) as compared to untreated cotton. Finally, the CO/CO_2_ ratio increases in the presence of the hybrid coating, hence indicating that it acts mainly in the condensed phase, favoring the formation of a stable protective char.

### 3.6. Durability (Washing Fastness)

For many applications, the resistance to laundry occasions is mandatory for flame-retardant fabrics. Therefore, some preliminary tests for assessing the washing fastness of the treated fabrics were performed according to the AATCC test method 61 (2A)–1996 on selected treated fabrics.

The data are collected in Table 8.

**Figure 13:**
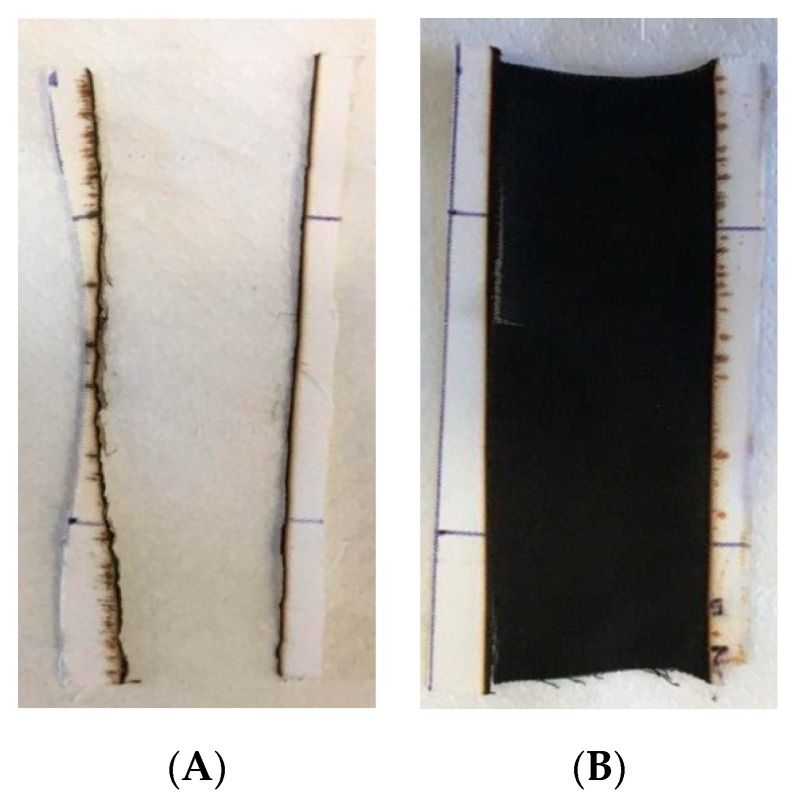
Residues after horizontal flame-spread tests for untreated cotton (**A**) and COT + SOL (70/30) (**B**) after washing treatment.

First of all, the high solubility of phytic acid in water causes the almost complete loss of the flame-retardant coating after washing, which results in significant weight losses for all the hybrid coated systems. COT + TEOS exhibits, in fact, the lowest weight loss after washing (13%). As a partial conclusion, these preliminary tests seem to indicate that the design hybrid coatings are not durable and therefore suitable for laundry occasions. In turn, this finding significantly affects the flammability of the treated fabrics, as shown in Table 9, which collects the results of the horizontal flame-spread tests performed on the washed fabrics. More specifically, all the treated fabrics, after washing, are not able to achieve self-extinction, though they still show some flame-retardant properties, witnessed by the lower burning rates and the higher final residues as compared to untreated cotton. It is possible to observe that, although the fibers burn completely (i.e., the applied flame propagates through the entire specimen length), the texture of the fabric is still maintained, and the residue is coherent (Figure 13). This finding indicates that the hybrid coating remained on the fabrics after washing still exerts a certain protection on the underlying fabric.

**Figure 14:**
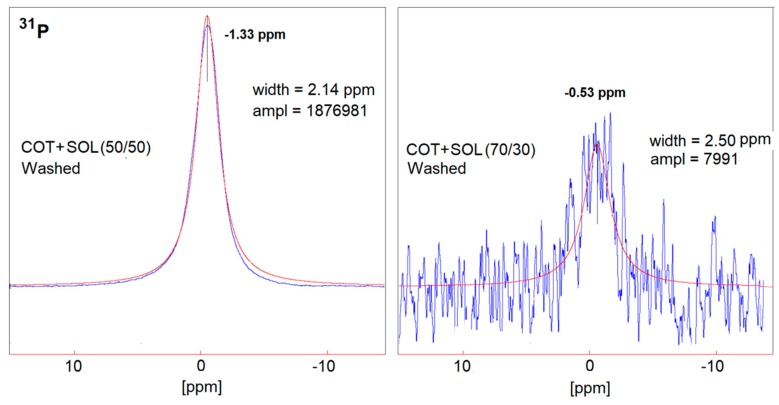
Solid state ^31^P (right) NMR spectra of COT + SOL (50/50) (left) and COT + SOL (70/30) (right) after washing.

**Figure 15:**
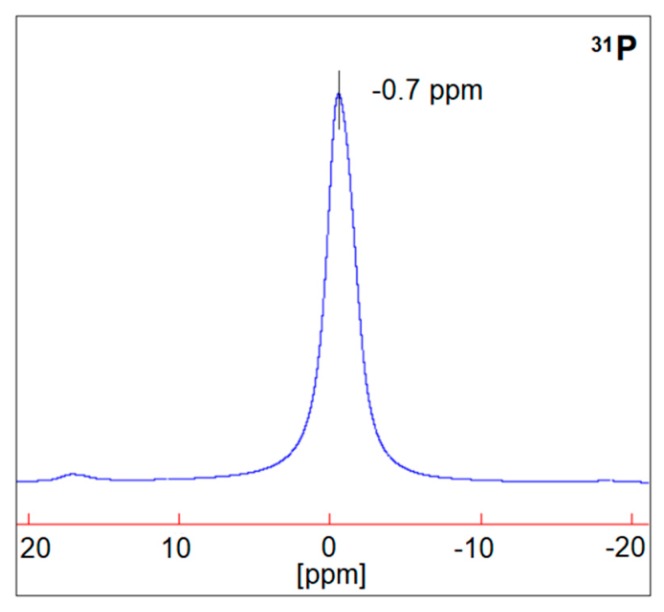
Solid state ^31^P NMR spectrum of the phytic acid solution.

**Figure 16:**
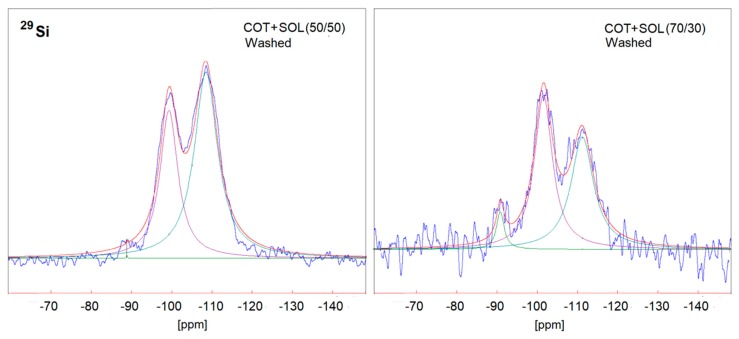
Solid state ^29^Si NMR spectra of COT + SOL (50/50) (left) and COT + SOL (70/30) (right) after washing.

In order to better understand the effect of the washing treatment on the structure of the hybrid coatings, solid-state NMR analyses were performed on selected treated fabrics (namely, COT + SOL (50/50) with 24% dry add-on and COT + SOL (70/30) with 16% dry add-on). First, it is noteworthy that the removal of phytic acid appears to be more severe in the case of COT + SOL (70/30), as indicated by the abrupt decrease in intensity of the ^31^P NMR peak (Figure 14), which is not observed in the case of COT + SOL (50/50) sample. By comparing the peaks before (Figure 4) and after washing (Figure 14), it can be seen that the structure of phytic acid is not affected by the washing process (^31^P NMR spectrum of the phytic acid solution is shown in Figure 15). Furthermore, considering the ^29^Si spectra of COT + SOL (50/50) sample shown in Figure 16, less reticulated Q^2^ species, detected in a small amount in the pristine sample (~0.5%), disappear after washing, while Q^3^ species slightly decrease (from 46.6% to 37.8%) in favor of Q^4^ (that rise from 56.3% to 62%). Conversely, the COT + SOL (70/30) sample exhibits a slightly different trend, with Q^3^ species becoming predominant after washing (Figure 16). However, beside such slight redistributions, no substantial difference can be detected, also taking into consideration that the peak intensities should not be taken as reliable indicators of the phase amount, as the dry add-on is higher for the COT + SOL (50/50) sample.

Finally, COT + SOL (70/30) with a dry add-on of 16% was subjected to cone calorimetry tests after washing; Table 10 compares the obtained results. It is worth noting that the partial washout of the hybrid coating worsens the fire behavior of the system, which approaches that of the untreated fabric, irrespective of the higher residue at the end of the test.

## 4. Conclusions

In the present work, new hybrid sol–gel systems (based on phytic acid and TEOS) were designed and applied to cotton fabrics, in order to study the effect of the deposited coatings on the thermal stability and flame-retardant properties of the cellulosic substrate.

FESEM and FTIR–ATR analyses confirmed the effective deposition of the coatings on the cellulosic substrate. Different TEOS/PA sols were prepared and applied to cotton; in any case, solid- state NMR analyses confirmed that the condensation reactions taking place during the sol–gel process occur with the formation of Q^2^, Q^3^, and Q^4^ species, leaving a very limited fraction of uncondensed molecules of the alkoxy precursor. Furthermore, these analyses demonstrated that phytic acid is not prone to react either with the hydroxyl groups of the cellulosic fabric, or with TEOS. A minimum total dry add-on of 16 wt % together with TEOS:PA ratio of 70:30 ensured self-extinction in both horizontal and vertical flame-spread tests. In addition, the hybrid coatings turned out to anticipate the thermal and thermo-oxidative degradation of cotton, favoring, at the same time, the formation of a stable char, which is able to act as a thermal shield, as revealed by the high residues found at the end of thermogravimetric analyses. Cone calorimetry tests pointed out, once again, that the sol–gel coatings anticipate the ignition of the samples, but, at the same time, are able to remarkably reduce HRR (−36%) and pkHRR (−75%), as well as to increase the final residues. Unfortunately, the flame-retardant properties were significantly lost after washing, as a consequence of the partial removal of the deposited hybrid coatings.

## Figures and Tables

**Table 1:** Composition of the treated cotton fabrics investigated.

Sample Code	Phytic Acid (wt %)	TEOS (wt %)	Dry Add-On A (wt %)
COT + PA	100	0	15
COT + SOL (40/60)	40	60	40
			33
			28
			24
COT + SOL (50/50)	50	50	30
			24
			21
			19
COT + SOL (60/40)	60	40	23
			19
			17
			14
COT + SOL (70/30)	70	30	19
			15
			13
			12
COT + TEOS	0	100	10

**Table 2:** Thermal stability of cotton and of the treated fabrics.

Atmosphere: Nitrogen				
Sample Code	*T*_ONSET_ (°C)	*T*_max1_ (°C)	Residue @*T*_max1_ (%)	Residue @700 °C (%)
COT	358	386	48.3	3.8
COT + PA	270	290	75.0	37.0
COT + SOL (40/60)	276	300	76.9	49.3
COT + SOL (50/50)	277	299	70.7	40.7
COT + SOL (60/40)	253	284	78.3	41.3
COT + SOL (70/30)	264	295	74.4	41.1
COT + TEOS	350	365	53.0	18.0

**Table 3:** Thermo-oxidative stability of cotton and of the treated fabrics.

Atmosphere: Air						
Sample Code	*T*_ONSET_ (°C)	*T*_max1_ (°C)	Residue@*T*_max1_ (%)	*T*_max2_ (°C)	Residue @*T*_max2_ (%)	Residue @ 700 °C (%)
COT	350	368	53.0	514	7.3	3.5
COT + PA	265	296	73.0	550	30.2	20.0
COT + SOL (40/60)	271	294	78.3	542	43.7	30.8
COT + SOL (50/50)	268	294	71.7	551	34.8	21.5
COT + SOL (60/40)	265	291	76.0	551	32.7	15.4
COT + SOL (70/30)	261	294	72.8	557	32.5	16.6
COT + TEOS	345	355	59.0	540	12.5	4.8

**Table 4:** Results of horizontal flame-spread tests.

Sample	Total Dry Add-On (%)	Self-Extinction	Residue (%)
COT	/	NO	0
COT + PA	8	YES	92
COT + SOL (40/60)	40	YES	89
	32	YES	87
	28	YES	86
	24	YES	85
COT + SOL (50/50)	30	YES	96
	24	YES	95
	21	YES	95
	19	YES	95
COT + SOL (60/40)	23	YES	97
	19	YES	96
	17	YES	96
	14	YES	95
COT + SOL (70/30)	19	YES	94
	16	YES	93
	13	YES	93
	12	YES	95
COT + TEOS	10	NO	11

**Table 5:** Results of vertical flame-spread tests.

Sample	Total Dry Add-On (%)	Self-Extinction	Residue (%)
COT	/	NO	0
COT + PA	8	NO	53
COT + SOL (40/60)	40	NO	77
	32	NO	74
	28	NO	60
	24	NO	55
COT + SOL (50/50)	30	YES	92
	24	YES	88
	21	NO	63
	19	NO	56
COT + SOL (60/40)	23	YES	96
	19	NO	68
	17	NO	55
	14	NO	56
COT + SOL (70/30)	19	YES	86
	16	YES	91
	13	NO	67
	12	NO	58
COT + TEOS	8	NO	5

**Table 6:** Combustion data for treated and untreated cotton fabrics. HRR is heat release rate, and THR is total heat release.

Sample	Time to Ignition (s)	HRR (kW/m^2^)	pkHRR (kW/m^2^)	Time to Peak (s)	THR (MJ/m^2^)	Residue (%)
COT	19	15.6	106.4	38	2.01	0
COT + SOL (70/30)	13	10.0	26.9	19	1.21	25.8

**Table 7:** Smoke parameters of treated and untreated cotton fabrics.

Sample	Total Smoke Release (m^2^/m^2^)	Total Smoke Production (m^2^)	Specific Extinction Area (m^2^/kg)	Mean CO Yield (kg/kg)	Mean CO_2_ Yield (kg/kg)	CO/CO_2_
COT	29.8	0.1	47.8	0.026	1.48	0.017
COT + SOL (70/30)	16.8	0.1	66.9	0.15	0.48	0.310

**Table 8:** Weight loss (%) of selected flame-retardant fabrics.

Sample	Weight Loss (%)
COT + PA	97
COT + SOL (40/60) Dry add-on = 33%	54
COT + SOL (50/50) Dry add-on = 24%	86
COT + SOL (60/40) Dry add-on = 19%	76
COT + SOL (70/30) Dry add-on = 16%	82
COT + TEOS	13

**Table 9:** Horizontal flame-spread tests performed on treated samples after washing.

Sample	Total Add-On (%)	*T*_1_ (s)	*T*_2_ (s)	*T*_tot_ (s)	Burning Rate (mm/s)	Residue (%)
COT	/	12	40	63	1.58	0
COT + PA	15	13	44	70	1.42	3
COT + SOL (40/60)	33	13	44	78	1.28	35
COT + SOL (50/50)	24	12	44	80	1.25	18
COT + SOL (60/40)	19	16	47	72	1.39	15
COT + SOL (70/30)	16	14	45	72	1.39	15
COT + TEOS	10	13	40	64	1.56	8

**Table 10:** Combustion data for treated cotton fabrics, before and after washing.

**Sample**	**Time to Ignition (s)**	**HRR (kW/m^2^)**	**pkHRR (kW/m^2^)**	**Time to Peak (s)**	**THR (MJ/m^2^)**	**Residue (%)**
COT + SOL (70/30)	13	10.0	26.9	19	1.21	25.8
COT + SOL (70/30) after washing	22	10.7	92.9	44	2.56	16.0
	**Total Smoke Release (m^2^/m^2^)**	**Total Smoke Production (m^2^)**	**Specific Extinction Area (m^2^/kg)**	**Mean CO Yield (kg/kg)**	**Mean CO_2_ Yield (kg/kg)**	**CO/CO_2_**
COT + SOL (70/30)	16.8	0.10	66.9	0.150	0.48	0.310
COT + SOL (70/30) after washing	15.8	0.05	29.8	0.063	1.59	0.040
